# PulmoNet: a novel deep learning based pulmonary diseases detection model

**DOI:** 10.1186/s12880-024-01227-2

**Published:** 2024-02-28

**Authors:** AbdulRahman Tosho Abdulahi, Roseline Oluwaseun Ogundokun, Ajiboye Raimot Adenike, Mohd Asif Shah, Yusuf Kola Ahmed

**Affiliations:** 1 Department of Computer Science, Institute of Information and Communication Technology, Kwara State Polytechnic, Ilorin, Nigeria; 2 Department of Multimedia Engineering, Kaunas University of Technology, Kaunas, Lithuania; 3 Department of Computer Science, Landmark University Omu Aran, Omu Aran, Nigeria; 4 Department of Statistics, Institute of Applied Sciences, Kwara State Polytechnic, Ilorin, Nigeria; 5 Department of Economics, Kebri Dehar University, Kebri Dehar, 250, Somali Ethiopia; 6 Centre of Research Impact and Outcome, Chitkara University Institute of Engineering and Technology, Chitkara University, Rajpura, Punjab 140401 India; 7 Chitkara Centre for Research and Development, Chitkara University, Baddi, Himachal Pradesh 174103 India; 8 Department of Biomedical Engineering, University of Ilorin, Ilorin, Nigeria; 9 Department of Occupational Therapy, University of Alberta, Edmonton, Canada

**Keywords:** Pulmonary diseases, Machine learning, Deep convolutional neural network, X-ray, CT scan, Accuracy

## Abstract

Pulmonary diseases are various pathological conditions that affect respiratory tissues and organs, making the exchange of gas challenging for animals inhaling and exhaling. It varies from gentle and self-limiting such as the common cold and catarrh, to life-threatening ones, such as viral pneumonia (VP), bacterial pneumonia (BP), and tuberculosis, as well as a severe acute respiratory syndrome, such as the coronavirus 2019 (COVID-19). The cost of diagnosis and treatment of pulmonary infections is on the high side, most especially in developing countries, and since radiography images (X-ray and computed tomography (CT) scan images) have proven beneficial in detecting various pulmonary infections, many machine learning (ML) models and image processing procedures have been utilized to identify these infections. The need for timely and accurate detection can be lifesaving, especially during a pandemic. This paper, therefore, suggested a deep convolutional neural network (DCNN) founded image detection model, optimized with image augmentation technique, to detect three (3) different pulmonary diseases (COVID-19, bacterial pneumonia, and viral pneumonia). The dataset containing four (4) different classes (healthy (10,325), COVID-19 (3,749), BP (883), and VP (1,478)) was utilized as training/testing data for the suggested model. The model’s performance indicates high potential in detecting the three (3) classes of pulmonary diseases. The model recorded average detection accuracy of 94%, 95.4%, 99.4%, and 98.30%, and training/detection time of about 60/50 s. This result indicates the proficiency of the suggested approach when likened to the traditional texture descriptors technique of pulmonary disease recognition utilizing X-ray and CT scan images. This study introduces an innovative deep convolutional neural network model to enhance the detection of pulmonary diseases like COVID-19 and pneumonia using radiography. This model, notable for its accuracy and efficiency, promises significant advancements in medical diagnostics, particularly beneficial in developing countries due to its potential to surpass traditional diagnostic methods.

## Introduction

Recently, the human race has been threatened by a high mortality rate due to pulmonary diseases. In the year 2020 alone, over 3.5 million pulmonary diseases such as Covid-19-related deaths were recorded worldwide, making it the third source of demise worldwide, with 80% of these demises, predominantly in developing and low to middle-income countries [1, 2]. Pulmonary disease is deadly because it affects the respiratory tissues and organs, especially the lungs, making breathing challenging for animals. Virtually all varieties of pulmonary disease share similar indications, including fever, dry cough, fatigue, and rapid shallow breathing [3].

Lung diseases posed enormous risk by causing around 4 million premature deaths annually until late 2019, when the risk factor doubled due to the outburst of the new coronavirus illness (Covid-19) in Wuhan, China [4, 5]. Since then, over 202 million cases have been reported, 4.2 million deaths, and an average daily cases of about 750,000 as of August 2021 [6]. Considering the transience degree owing to lung ailment infections and the contagious nature of some of these diseases, coupled with time-consuming and expensive diagnosis procedures, there is an urgent need for responsive diagnostic and detection systems, as early detection could save many lives by preventing premature deaths.

One promising approach to the automated diagnosis of illnesses recently is artificial intelligence (AI) [7–9]. It has been revolutionizing the medical and healthcare industries in many ways, such as drug research and medical imagery, with a reasonable level of accuracy [10]. Two major techniques embody AI: ML and deep learning (DL). It has shown its efficacy by solving problems ranging from natural language processing to image classification using different DL and ML models. It makes predictions and inferences by analyzing the large quantity of input data, performs intelligent tasks such as feature detection, pattern recognition, translation, and perceptron on the data, and then makes the relevant decision [11–13]. Because of the high performance and other proven efficient qualities of AI in image identification and classification tasks, many researchers have conducted studies linking AI-founded techniques to the classification and prediction of different pulmonary diseases using either CT or X-ray imageries [14–17].

The challenge of possible overlap of symptoms of pulmonary diseases makes diagnosis difficult, especially when there is a shortage of experienced personnel or an absence of a patient’s health record at hand. This scenario prompted for creation of an automated diagnosis process that can accurately detect the presence of any of the three pulmonary diseases earlier mentioned. To the authors’ knowledge, this investigation is the first to utilize AI techniques to classify and detect three pulmonary diseases.

For this reason, this investigation, therefore, suggested a DCNN-based image detection model, specifically, a CNN optimized with an image augmentation approach, to detect three (3) different pulmonary diseases (COVID-19, BP, and VP). CT and X-ray imageries were utilized to have more image datasets, enough to make concrete inferences and enhance the model’s detection ability, as CT can detect abnormal features in the chest images even before symptoms appear.

The core contribution of this study is as follows:

1. Proposed a novel PulmoNet model based on the DCNN model capable of performing multiclass image classification utilizing CT scans and X-ray images.
2. Use of an image augmentation technique to improve the proposed DCNN model
3. Use of DCNN models to classify and detect three pulmonary diseases.
4. Classification of pulmonary diseases into four (4) classes, three (3) classes, two (2) classes (Covid_19 and Healthy), and two (2) classes (Pneumonia and Healthy).
5. Evaluation of the proposed system using confusion matrix performance measures such as precision, recall, and f1-score
6. Comparison of the suggested PulmoNet model with existing systems

The research aimed to create and evaluate a specialized deep convolutional neural network (DCNN) model for accurately detecting various lung illnesses by analyzing radiographic imaging methods, such as X-rays and CT scans. The focus of our study was on carefully constructing and perfecting this model. The process included thorough assessments of the model’s accuracy in detecting illnesses such as COVID-19, bacterial pneumonia, and viral pneumonia. In addition, the research sought to compare the diagnostic capabilities of the DCNN model with those of traditional approaches to acquire a thorough grasp of its effectiveness and future enhancements in the area of medical diagnostics.

The remainder of this article is prearranged thus: “Related literature” section emphasizes the analysis of related literature; “Methodology” section discusses the research methods, including the materials, various datasets, and their repositories. “Results and discussion” section demonstrates the experimented outcomes and discussion, and lastly, “Discussion” section concludes the investigation.

## Related literature

Several research works have experimented with AI in healthcare to improve the conventional healthcare system and industry [18]. The need for an affordable, efficient and accurate diagnostic system that can serve as an alternative to the existing traditional time-consuming and expensive diseases diagnosis, especially pulmonary diseases, triggered an upsurge in AI-in-healthcare research [19–21]. To investigate the relationship between AI schemes and diseases detection, researchers have conducted investigations ranging from predicting future diseases using patient’s previous historical health records to detecting and identifying possible ailments using CT and X-ray imageries [10, 15, 22, 23].

Due to the similarity of symptoms of pulmonary diseases, experts sometimes misdiagnose one for the other [24]. For instance, when the coronavirus 2019 (COVID-19) pandemic broke out officially in Wuhan, China, around December 2019 [1, 25–27], it was initially thought to be severe acute pneumonia, probably severe acute respiratory syndrome (SARS) or middle east respiratory syndrome (MERS) until it was established as novel COVID-19. Because of its novelty, there were challenges in testing and diagnosis as it is highly infectious. Many health workers have been unconsciously infected while the medical services provisioning and the testing kits were either unavailable or expensive and time-consuming [14]. This necessitates an alternative method of virus detection, of which AI has shown a great prospect, as pointed out by Haenssle et al. [25]; Mar & Soyer [28].

Radiological images obtained from CT and X-rays have similarly been a great source of data for training AI-founded disease recognition and analysis systems to achieve swift pulmonary disease diagnosis and management [29]. CT and X-ray imageries have been identified as sensitive means of detecting pneumonia in COVID-19 patients and can be exploited as screening equipment sideways with reverse transcription-polymerase chain reaction (RT-PCR), the utmost popular COVID-19 recognition technique [30]. In Pan et al. [30], changes were observed in COVID-19 pneumonia survivors’ lungs as revealed by CT scan imageries ten days after the start of the indications. Chan et al. [31] also pointed out that changes were observed in CT and X-ray imageries of the chest long before clinical symptoms of COVID-19 appeared. These changes can vary from right infrahilar space opacity, as in Kong & Agarwal [32]), to round lung opacities Zu et al. [33] and ground-glass opacities (GGO) or mixed GGO in so many patients [33]. Furthermore, it was identified that one out of three patients studied had one nodular opacity at the lung’s lower left region, whereas others had up to four and five irregular opacities in the two lungs [34–36].

Of all the AI schemes that have been adopted in healthcare systems, DL-based techniques have been promising [37–39]. One DL-based method popularly adopted for various disease recognition exploiting CT scans and/or X-ray imageries is CNN [40, 41]. It is biologically inspired as its neuron connectivity pattern resembles the animal visual cortex [42, 43]. CNN is the best commonly used technique for image categorization because it relatively optimizes the kernels via automated learning and requires little preprocessing [44, 45].

CNN Pulmonary disease detection systems have been well-researched in recent years due to the COVID-19 global pandemic. Although so many excellent research works on using CT [46] and X-ray imageries to detect pulmonary diseases have been published, they are not yet an alternative to the traditional methods of diagnosis. However, they have promised to be a helpful association with the traditional diagnosis methods, thereby creating a huge avenue for research and amelioration before commercialization. (Some of the research works focusing on DL-based pulmonary diseases detection using features from CT imageries and X-ray imageries of in-patients include the work of [47]. In their work, a deep feature network, specifically ResNet50, was combined with a support vector machine (SVM) to identify patients infected with COVID-19 and classify normal patients from infected ones. Although their work achieved an accuracy of about 95.4%, it only focused on a single class detection (COVID-19). Similarly, Hemdan et al. [48] proposed a DL-based classifier (COVIDX net) to categorize two classes of 50 clinical X-ray imageries of COVID-19 and normal chest images. Although their model accomplished about 90% accuracy, the dataset utilized to train and test the suggested techniques is not enough for the model to generalize.

In another work, Song et al. [49] established a classifier (DRE-Net) for COVID-19 and non-COVID-19 categorization utilizing CT imageries. The resulting technique has an accuracy of about 86% but only focuses on two-class classification with only 88 CT scan images. Also, in Wang et al. [50], a two-class classification system was suggested using CT scan images. An M-inception model was modified and tested on the obtained pathogen-confirmed COVID-19 images, and the resulting model had an average of about 84.4% accuracy.

Apart from these previous works, other research focuses on more than two classes of pulmonary disease detection and classification. Some of these works include Narin et al. [51], which suggested a DCNN ResNet-50 technique to categorize four classes of (COVID-19, normal (healthy), VP, and BP). The technique accomplished an accuracy of about 98% for two-class classification. Also, Ozturk et al. [52] proposed that the DarkCovidNet model performs two and three-class classifications, and the technique achieved an average accuracy of about 90.6% for both class classifications. Khan et al. [53] proposed that Xception-base Coronet was exploited for multiclass categorization by exploiting X-ray imageries.

This study focuses on building, training, and evaluating the effectiveness of the suggested PulmoNet founded on the CNN model and capable of performing multiclass image classification exploiting CT scans and X-ray imageries. It comprises a combination of convolutional, dense, max pooling, and flattened layers combined with four (4) different activation functions: ReLU, Sigmoid, Leaky ReLU, and Tanh.

The current body of research in artificial intelligence (AI) and healthcare primarily emphasizes the creation of cost-effective, streamlined, and precise diagnostic tools, with a particular emphasis on pulmonary disorders. Research encompasses a wide range of activities, including predicting illnesses based on historical health information and using CT and X-ray images for disease diagnosis. There is a strong focus on the need for alternative approaches because of the incorrect identification of comparable symptoms in respiratory disorders, which was brought to attention during the COVID-19 pandemic. Research has used radiological images to teach artificial intelligence systems, demonstrating their efficacy in rapid diagnosis. Deep Learning (DL) methods, particularly Convolutional Neural Networks (CNN), have been significant in these investigations, with CNN being a favoured option because of its practical picture classification skills. Several studies have investigated deep learning (DL) algorithms for identifying lung illnesses utilizing CT and X-ray images, with specific emphasis on detecting COVID-19. These investigations have shown encouraging outcomes. However, they have not reached comparability with conventional diagnostic techniques. This suggests that further study and development are necessary.

## Methodology

The methodology employed to achieve the aim and objectives of this work is in three stages, which include:

- Data collection
- Data preprocessing and splitting
- Model design, training, and evaluation

### Data collection

**Fig. 1:**
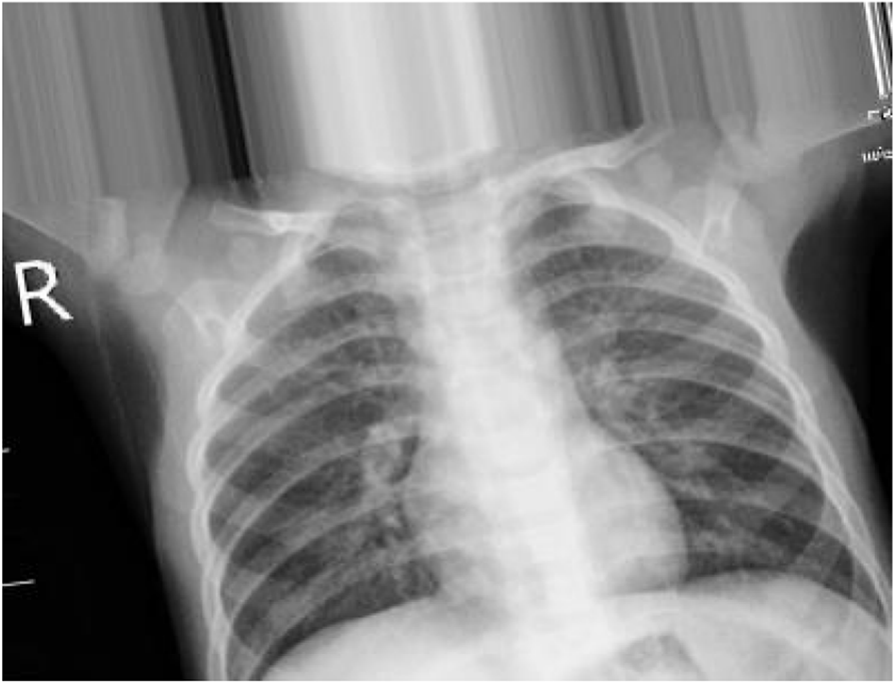
Sample of chest X-ray image of a BP patient

**Fig. 2:**
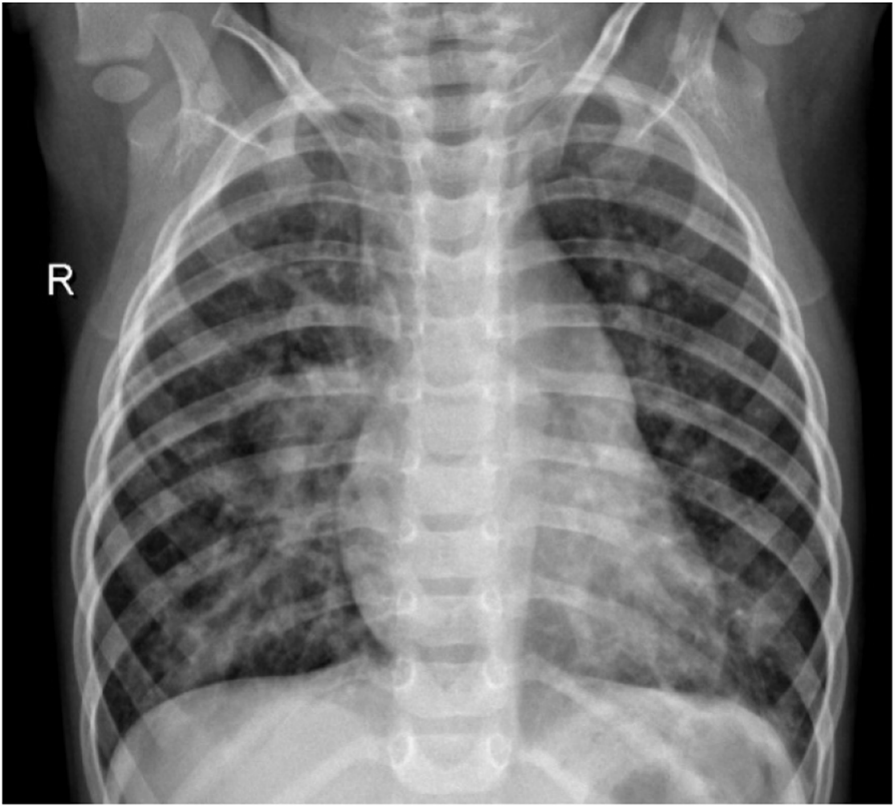
Sample of chest X-ray image of a non-Covid VP patient

**Fig. 3:**
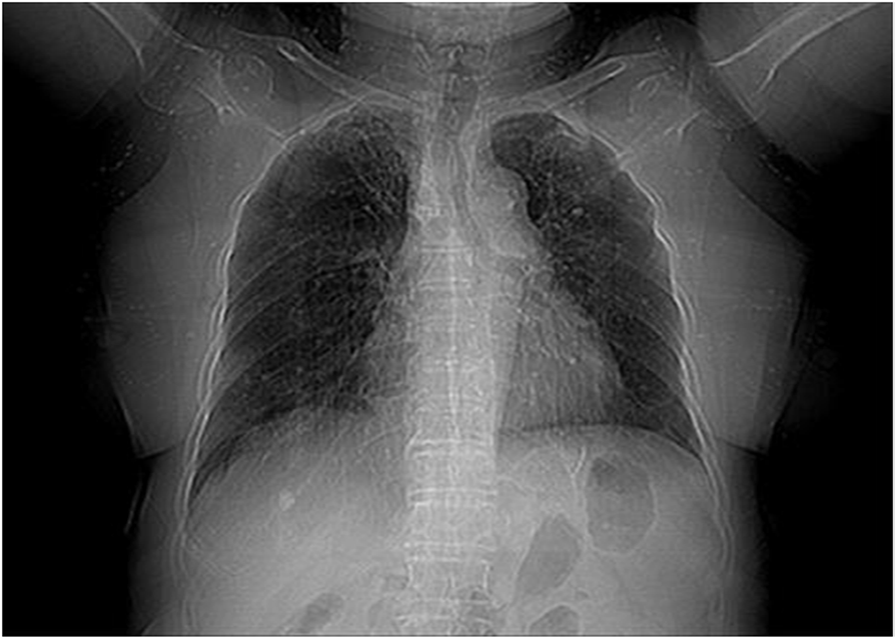
Sample of chest X-ray image of a Covid-19 patient

**Fig. 4:**
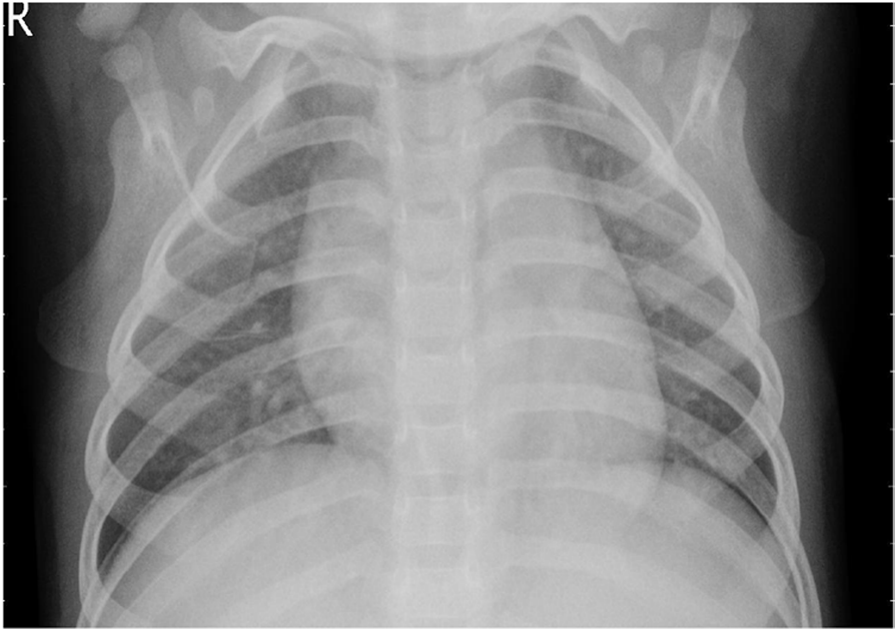
Sample of chest X-ray image of a healthy person

The dataset for this investigation was obtained from the combination and modification of different Covid-19, VP, BP, and healthy data repositories. These include the Actualmed-covid-chest x-ray dataset [54], SARS-COV-2 CT-scan dataset [55], x-ray dataset of Covid-19 Pneumonia detector and Covid-19 Radiography Database on Kaggle [56, 57], Covid-19 image dataset of Cohen et al. [55] and so on. In the end, 16,435 image datasets comprised of 883 bacterial pneumonia images, 1,478 viral pneumonia images, 3,749 covid-19 images, and 10,325 healthy images were generated. Sample imageries for each of the classes are presented in Figs. 1, 2, 3 and 4.

Figure 1 presents an x-ray imageries of a BP patient. The figure shows a focal opacity that indicates consolidation without cavitation on the right lung’s upper lobe. The cardio-phrenic and costo-phrenic angles can be seen to be free of air-fluid level.

Figure 2 presents an x-ray image of a non-Covid VP patient. The image demonstrates extended two-sided ground glass opacities (GGO), which indicates extended septal thickenings and alveolar mutilation. Similarly, in Fig. 3, the chest x-ray of a Covid-19 patient is presented. The radiography image shows hyperlucent lung fields, which specify lung hyperinflation owing to such small airway obstruction caused by bronchitis. Other features, such as manifold patchy opacities in both lung fields and blunting of costo-phrenic angles, can also be observed in the image.

Figure 4 presents the x-ray representation of a healthy person. The radiography chest image shows the normal shape and size of the chest wall in a normal situation and with a normal presence of the trachea, mediastinum, and heart. No focal opacity, air-fluid level, abnormal bronco-vascular markings, or hyperlucency is observed. Both cardio-phrenic and costo-phrenic angles are also unrestrained.

### Description of dataset

The dataset used for the study consists of 16,435 image datasets which are divided into four categories: bacterial pneumonia, viral pneumonia, COVID-19, and healthy cases. Here are the details of the dataset:

- Bacterial Pneumonia (BP): 883 images.
- Viral Pneumonia (VP): 1,478 images.
- COVID-19: 3,749 images.
- Healthy: 10,325 images.

These images were sourced from various public data repositories and modified to create a comprehensive dataset for the study. Notably, the repositories included the Actualmed-COVID-chest x-ray dataset, SARS-COV-2 CT-scan dataset, a COVID-19 Pneumonia detector x-ray dataset, and the COVID-19 Radiography Database available on Kaggle. Additionally, the COVID-19 image dataset from Cohen et al. was also utilized.

The images for each class were presented in the study to demonstrate typical features associated with each condition. For example, the BP patient images showed focal opacity without cavitation in the right lung’s upper lobe, indicative of bacterial pneumonia.

For the model training and testing, the dataset was divided into 85% for training and 15% for testing. The dataset’s distribution across various classes was instrumental in training the model to distinguish between the different pulmonary diseases and healthy cases, demonstrating the effectiveness of the model in terms of accuracy and potential for real-world application.

The comprehensive dataset was vital in achieving the high detection accuracies reported in the study: 94% for 4-class classification, 95.4% for 3-class, 99.4% for COVID-19 vs. Healthy, and 98.3% for Pneumonia vs. Healthy. These results indicate the model’s proficiency compared to traditional pulmonary disease recognition methods using x-ray and CT scan images.

### Data preprocessing and splitting

After the dataset is generated, the sizes of x-ray images are reduced by rescaling to obtain a faster model training process. The rescaled images are then converted to greyscale. The obtained preprocessed dataset is then subdivided into three subsets for 4 class, 3 class, and 2 class classifications correspondingly. Tables 1, 2 and 3 show the detail of each class classification and the sum of images utilized for training and testing each class. Finally, the dataset is split into 85% training and 15% testing samples.

**Table 1:** Summary of the parameters used for the PulmoNet model

Hyperparameters	Value
Input shape	(64, 64, 1)
Number of Layers	26
Loss Function	Categorical Cross_entropy
Learning Rate	0.001
Optimizer	Adam
Activation Functions	ReLU, Softmax
Performance Metrics	Accuracy, precision, recall and f1-score

**Table 2:** Accuracy per class (4 classes)

Class	ACC	Number of samples
Bacterial_Pneumonia	95	131
Covid_19	93	561
Viral_Pneumonia	91	221
Healthy	97	1,548

**Table 3:** Accuracy per class (3 classes)

Class	ACC	Number of samples
Bacterial_Pneumonia	95	131
Covid_19	96	561
Healthy	95.3	395

Significant disparity in the number of input images for each class have been handled using image augmentation method as it is a valuable strategy for addressing data imbalance in CNN models. By artificially expanding the training dataset with augmented samples, it helps to alleviate the challenges posed by imbalanced class distributions. Various image augmentation techniques employed to introduce diversity and variability into the training data and in turn improve the detection ability of our model include:

1. Rotation: Rotating images within a certain range to simulate different angles of view.
2. Translation: Shifting images horizontally or vertically to simulate different positions within the frame.
3. Scaling: Rescaling images to different sizes, allowing the model to learn robustness to variations in object sizes. And
4. Flipping: Mirroring images horizontally or vertically to create additional variations.

### Model design, training, and evaluation

This study’s proposed model (PulmoNet) is an improved 26-layer CNN-based model motivated by a wide residual network (WRN) architecture. This version of CNN was considered because it takes a shorter training time due to its shallow nature. WRN solves problems faster by adding shortcuts between its various layers, thereby preventing distortion that can occur as the network becomes complex. The model was implemented using Keras, Tensorflow, and Jupyter notebook. The training was done using an NVIDIA K80 graphics processing unit (GPU) with and batch size of 16, an epoch of 50, a learning rate of 0.001, and Adam optimizer were set as training parameters. Batch normalization was employed to stabilize the technique by standardizing the input size. Various hyperparameter combinations experimented with convolution, and max pool layers gradually increased as data shuffling was enabled at each epoch. The effectiveness of the examined approach was observed and recorded each time these layers were increased until a better and more stable outcome was recorded. All these were done to obtain a sensitive approach that can obtain any slight change in features of the input images.

In the end, the final model consists of 26 layers in total, including convolutional layers (‘Conv2D’), max-pooling layers (‘MaxPooling2D’), a flatten layer, and densely connected layers (‘Dense’). The activation function used is ReLU, except for the output layer where softmax activation is used for multi-class classification. The model is compiled with the Adam optimizer and categorical cross-entropy loss. Summary of parameters of proposed PulmoNet model is presented in Table 1. It can be demonstrated that the model input images with input shape (64, 64, 1) and loss function used for the implementation is Categorical Cross_entropy. A learning rate of 0.001 was set for the experiment with adam optimizer. The activation functions used was ReLU and Softmax at the dense layer. While training the model the metric used was accuracy.

An n-fold cross-validation technique was exploit to assess the effectiveness of the suggested classification technique by randomly dividing the training dataset into three (3) equal parts. Two were utilized for training, and the remaining was utilized for validation. Shifting of the testing and validation sets was strategically repeated continuously Five (5) times. The overall performance of the suggested approach was assessed by computing the average values from each fold.

Finally, the suggested approach’s effectiveness was evaluated by exploiting a system of measurement which includes accuracy (Acc), precision (Prec), recall (Rec), f1-score, and confusion matrix.

### PulmoNet mathematical background

PulmoNet is a deep CNN-based model developed to classify multiclass images using CT scans and X-ray images. It is a 26 layers model motivated by WRN architecture which introduces the concept of widening the network by increasing the number of channels in the convolutional layers compared to traditional Residual Networks (ResNets). The key idea is to have a wider network with an increased capacity to capture more complex patterns. An overview of the mathematical representation of PulmoNet is presented thus:

Assuming input images of size (W, H, C), where W is the width, H is the height, and C is the number of color channels.

And convolutional layers performing convolution operation such that:

- Each convolutional layer applies a set of filters to the input image.
- Mathematically, the convolution operation can be defined as:

1 $$C\_i=A\_i\left(Conv2D\left(F\_i,K\_i\right)\left(C\_\left\{i-1\right\}\right)\right)$$

where *C_i* is the output feature map of the *i-th* convolutional layer, *F_i* is the filter/kernel of size *K_i*, and *A_i* is the activation function applied to the output.

The main building blocks of the WRN are the Wide Residual Blocks, which contain multiple convolutional layers and skip connections.

Each Wide Residual Block consists of the following operations:

- Batch Normalization which normalizes the activations of the previous layer.
- Activation which applies an activation function, such as ReLU.
- Convolution which applies a series of convolutional layers with increased channel size. And
- Skip Connection which adds the input of the block to the output of the block, creating a shortcut connection.

Mathematically, the Wide Residual Block is represented thus:

2 $$B\_i=SkipConnection\left(BatchNormalization\left(Activation\left(Convolution\left(F\_i,K\_i\right)\right)\right)\right)+C\_\left\{i-1\right\}$$

Where *B_i* is the output of the *i-th* Wide Residual Block and $C\_\left\{i-1\right\}$ is the input of the block.

After the Wide Residual Blocks, a global average pooling operation is performed to aggregate the spatial information into a single vector.

Mathematically, the global average pooling operation is represented as:

3 $$GAP=GlobalAveragePooling2D()\left(B\_n\right)$$

Finally, a fully connected layer is applied to process the aggregated features.

It is Mathematically represented as thus:

4 $$D=Dense\left(U,activation=A\right)\left(GAP\right)$$

The softmax activation function is applied to the output layer to obtain class probabilities and it is represented mathematically as:

5 $$S=Soft{\text{max}}\left(D\right)$$

Categorical cross-entropy loss function is then applied and represented mathematically as thus:

6 $$L=CategoricalCrossentropy\left(S,True\_Labels\right)$$

During training, the model aims to minimize the loss by adjusting its weights and biases and Adam optimizer is applied to update the model’s parameters.

Mathematically, the parameter update is represented as:

7 $$\theta \_i=\theta \_i-learning\_rate*\partial L/\partial \theta \_i$$

Where $\theta \_i$ represents the trainable parameters in the *i-th* layer, learning_rate is the learning rate for the optimizer, and $\partial L/\partial \theta \_i$ is the derivative of the loss with respect to $\theta \_i$.

The provided description outlines the mathematics behind the operations of a Convolutional Neural Network (CNN), particularly focusing on Wide Residual Networks (WRN). These equations and operations collectively constitute the forward and backward passes of training a CNN, enabling it to learn from data and make predictions.

1. *Convolution Operation (Eq. *1*)*: This describes the process where a set of learnable filters is applied to the input image to create feature maps. Each filter detects different features, and an activation function like ReLU introduces non-linearity, helping the network learn complex patterns.
2. *Wide Residual Blocks (Eq. *2*)*: WRNs improve upon standard residual networks by increasing the width (number of channels) instead of the depth (number of layers). These blocks use batch normalization for stabilizing learning, and skip connections for alleviating the vanishing gradient problem by allowing direct gradients flow.
3. *Global Average Pooling (Eq. *3*)*: This operation reduces each feature map to a single number by averaging out the spatial dimensions, reducing the total number of parameters and computation in the network.
4. *Fully Connected Layer (Eq. *4*)*: Here, the pooled features are flattened and connected to as many neurons as there are classes, to prepare for classification.
5. *Softmax Function (Eq. *5*)*: This activation function is used in the output layer to calculate the probabilities of each class, ensuring they sum up to one.
6. *Categorical Cross-Entropy (Eq. *6*)*: It’s a loss function suitable for multi-class classification problems, measuring the difference between the predicted probabilities and the actual class.
7. *Parameter Update (Eq. *7*)*: Represents the optimization process, where the Adam optimizer is typically used to adjust the weights and biases to minimize the loss function.

### Algorithm of the proposed model

Algorithm: PulmoNet for Pulmonary Disease Detection

Input: Set of labeled radiographic imaging data (X-rays/CT scans)

Output: Disease classification (e.g., Healthy, Bacterial Pneumonia, Covid_19, Viral Pneumonia)

Begin

1. Data Collection:
  Collect diverse datasets of labeled images.
2. Data Preprocessing:
  Perform image augmentation to enhance dataset size and variability.
  Normalize images and convert to grayscale if necessary.
  Split data into training, validation, and test sets.
3. Model Design (PulmoNet using CNN):
  Define a CNN architecture with layers suitable for image analysis.
  Apply convolutional layers with filters to extract features.
  Utilize activation functions like ReLU for non-linearity.
  Incorporate Wide Residual Blocks for deeper learning with fewer issues.
  Use Global Average Pooling to reduce dimensionality.
  Add Fully Connected Layers for classification.
  Implement softmax activation for output layer probability distribution.
4. Model Training:
  Use categorical cross-entropy as the loss function.
  Apply Adam optimizer for efficient training.
  Train model using backpropagation and mini-batch gradient descent.
5. Model Evaluation:
  Evaluate model using accuracy, precision, recall, and F1 score on validation set.
  Adjust hyperparameters and model structure based on performance.
6. Model Testing:
  Test the final model on unseen data.
  Generate a confusion matrix to understand classification performance.

End

## Results and discussion

The outcomes of implementing the proposed approach for the 4 class, 3 class, and 2 class classifications are calculated and presented. Performance of the model for each class after training and validation procedures are evaluated in terms of accuracy (acc), precision (prec), recall (rec), f1 score, and confusion matrix.

The suggested approach acc was calculated as the ratio of the sum of images categorized correctly by the model to the entire sum of all images. Mathematically,

8 $$Acc= \frac{Number\ of\ images\ classified\ correctly}{Total\ number\ of\ all\ images}$$

Results of accuracy per class for all three (3) classes were presented in Table 1, 2, 3 and 4.

**Table 4:** Accuracy per class (2 classes)

Class	ACC	Number of samples
Covid_19	99	561
Healthy	99.8	395

The tables in this section present the accuracy of the PulmoNet model in classifying pulmonary diseases across different class configurations. Table 2 presents the diagnostic accuracy of the PulmoNet model for four different classes based on the data. The diagnostic tool has exceptional precision in differentiating between various lung diseases, with a 95% accuracy rate for bacterial pneumonia, 93% for COVID-19, and 91% for viral pneumonia. The model demonstrates the highest level of precision in correctly identifying persons in good health, with an accuracy rate of 97%. The varied samples for each class show the model’s resilience across diverse dataset sizes, ranging from 131 for bacterial pneumonia to 1,548 for healthy.

The PulmoNet model has robust diagnostic accuracy for three categories, as shown in Table 3. The model accurately detects bacterial pneumonia in 95% of cases out of 131 samples, identifies COVID-19 in 96% of cases out of 561 samples, and correctly diagnoses healthy instances with an accuracy of 95.3% out of 395 samples. This demonstrates consistent and dependable performance in both illness and healthy conditions, suggesting that the model might be efficiently used within these three categories for screening purposes.

Table 4 demonstrates the remarkable efficacy of the PulmoNet model in a binary classification test. It achieved a 99% accuracy in detecting COVID-19 from a dataset of 561 samples and an almost perfect accuracy of 99.8% in recognizing healthy patients from a dataset of 395 samples. These statistics indicate that the model is proficient in differentiating between COVID-19 and healthy lung conditions, demonstrating its potential as a highly dependable instrument for rapid screening in medical environments.

Table 5 displays the accuracy of the model in a binary classification test, specifically in differentiating between occurrences of pneumonia and healthy patients. The model has a remarkable discriminative ability in this binary classification scenario, accurately detecting pneumonia with a 97% precision from a sample size of 131 and identifying healthy patients with a 99% precision from a sample size of 395.

**Table 5:** Accuracy per class (2 classes)

Class	ACC	Number of samples
Pneumonia	97	131
Healthy	99	395

As shown in the Tables, different prediction accuracy was recorded per class. In Table 2, our model recorded an accuracy of 97% for healthy, 95% for bacterial pneumonia, 93% for covid-19, and 91% for viral pneumonia in the 4-class classification. In Table 3, the accuracy for healthy images reduced to 95.3%, and that of covid_19 improved to 96%, while that of bacterial pneumonia is 95% for the 3-class classification. Tables 4 and 5 are for 2 class classifications but for different class combinations. It can be seen that accuracies of 99% and 99.8% were recorded for both covid_19 and healthy, while 97% and 99% were recorded for pneumonia and healthy.

**Fig. 5:**
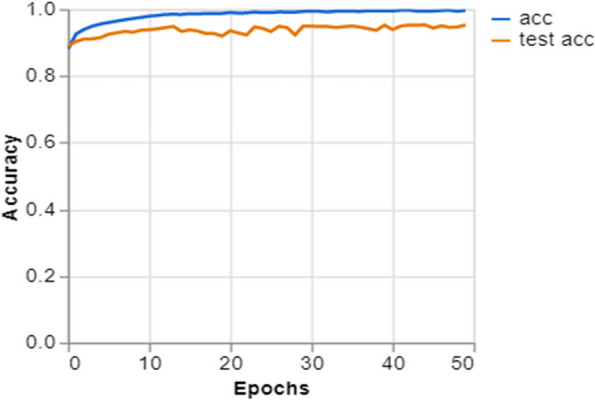
Model Accuracy per epoch for 4 class classification

**Fig. 6:**
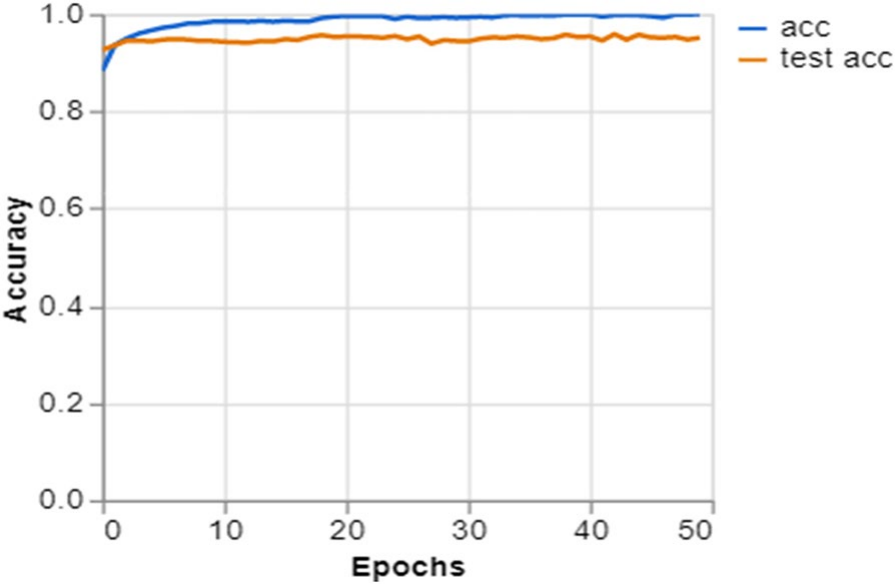
Model Accuracy per epoch for 3 class classification

**Fig. 7:**
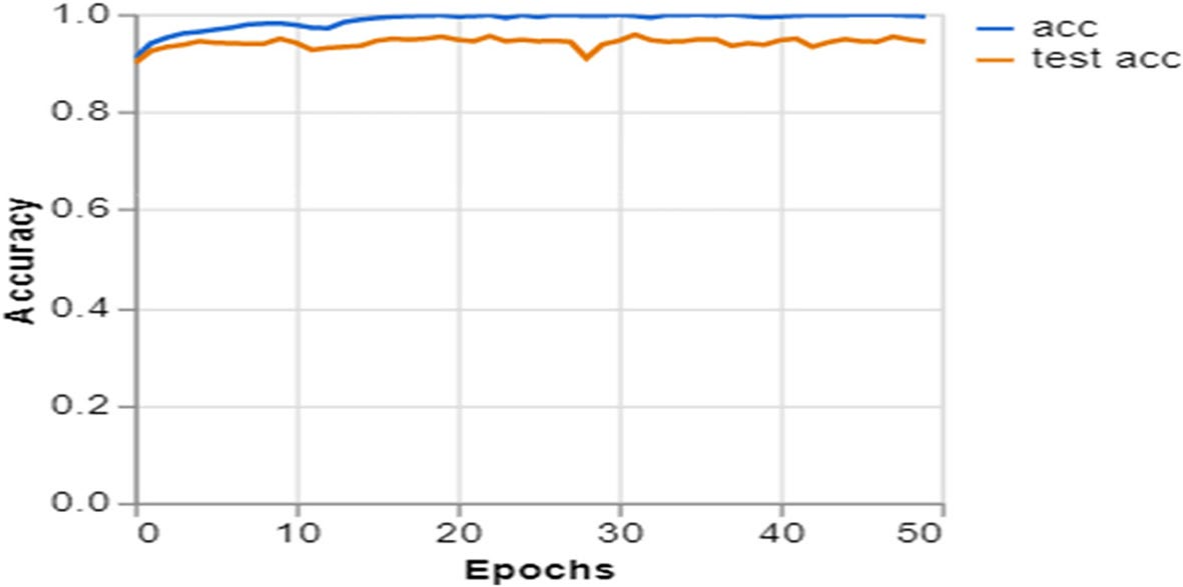
Model Accuracy per epoch for 2 class classifications (Covid_19 and Healthy)

**Fig. 8:**
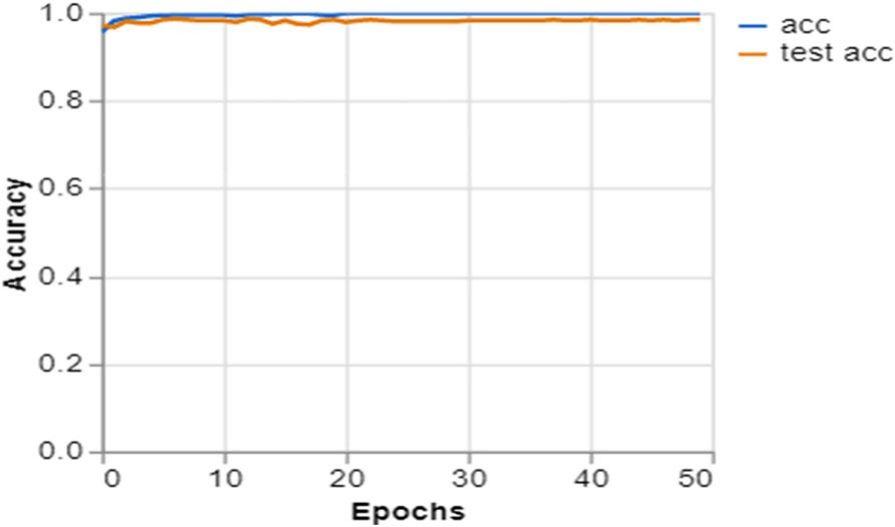
Model Accuracy per epoch for 2 class classifications (Pneumonia and Healthy)

**Fig. 9:**
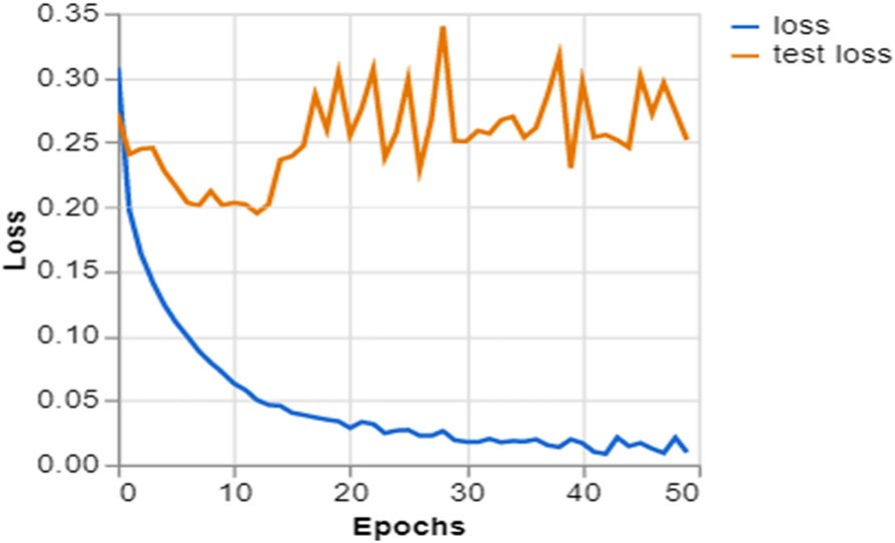
Model loss per epoch for 4 class classification

**Fig. 10:**
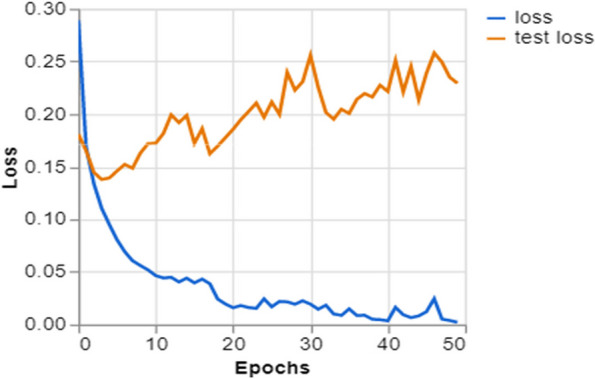
Model loss per epoch for 3 class classification

**Fig. 11:**
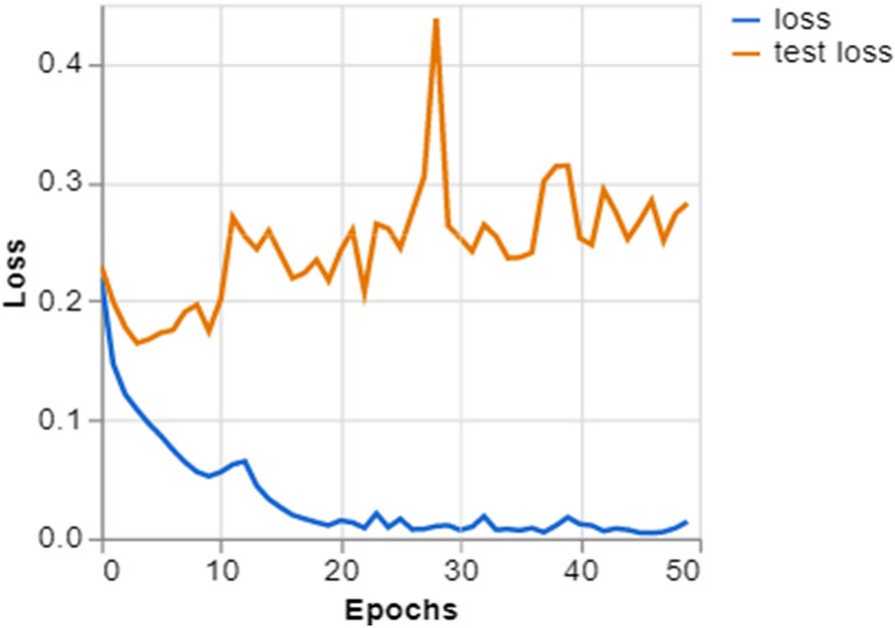
Model loss per epoch for 2 class classifications (Covid_19 and Healthy)

**Fig. 12:**
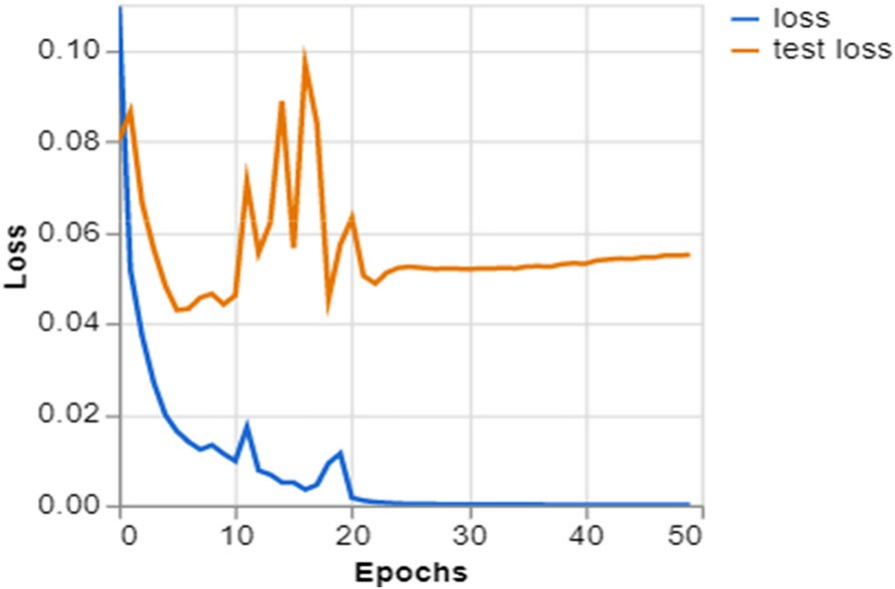
Model loss per epoch for 2 class classifications (Pneumonia and Healthy)

To further demonstrate how well our model performed, graphs of model accuracy and loss per epoch for each class classification are presented in Figs. 5, 6, 7, and 8. Furthermore, the confusion matrix for each class classification is also obtainable in Figs. 9, 10, 11, and 12. These Figures indicate that the suggested model is well-trained without over-fitting or under-fitting, and this is further justified by the obtained results of precision, recall, and f1 score, respectively.

The Fig. 5 illustrates the accuracy trends across epochs throughout the training and validation phases of a deep learning model. The training accuracy (acc) is represented by the blue line, while the validation accuracy (test acc) is shown by the orange line. Both accuracies exhibit fast improvement during the early epochs, suggesting that the model is effectively acquiring knowledge from the data. The model reaches a state of convergence near the end, indicating that it no longer gains significant knowledge from further training. The proximity of the two lines indicates strong generalization, since the validation accuracy closely corresponds to the training accuracy, indicating that the model is not excessively fitting to the training data.

Figure 6 depicts the progression of a deep learning model’s performance throughout the training process over a fixed number of epochs for a job that involves classifying data into three distinct categories. The two lines, denoting training accuracy (acc) and validation accuracy (test acc) indicate the model’s proficiency in learning and generalizing to novel data. The elevated numbers suggest the model performs strongly on the training and unseen validation data. The precise alignment of the lines with minimal deviation indicates that the model does not excessively fit the data and has successfully acquired the ability to generalize the distinguishing characteristics of the three classes. This shows that the model is well-calibrated and has the potential to be dependable in a real-world scenario for identifying the provided classes, which may include different types of pulmonary disorders.

Figure 7 illustrates the progression of a deep learning model in accurately classifying two classes. The accuracy trends for the training set (acc) and validation set (test acc) increase rapidly in the beginning epochs and subsequently level off, remaining at high levels around 1.0, indicating almost flawless accuracy. This plateau demonstrates that the model has successfully acquired the ability to differentiate between COVID-19 and healthy categories. The proximity between the training and validation accuracy suggests that the model effectively applies to unfamiliar data, a crucial attribute for reliable diagnostic tools in clinical environments.

Figure 8 displays the accuracy of a machine learning model during training (acc) and validation (test acc) as it learns to differentiate between pictures of lungs with pneumonia and healthy lungs. The training and validation accuracies of the model reach a high level and are stable early in the training phase, indicating a rapid capacity to learn and generalize. The consistent and steady accuracy in the training and validation phases suggests that the model is appropriately calibrated and does not suffer from overfitting. This is crucial for ensuring the model’s reliability in real-world medical diagnostics. The graph indicates that the model has the potential to be a valuable tool for screening and diagnosing pneumonia based on radiographic data.

Figure 9 shows the loss of the model for the training data (loss) and validation data (test loss) across 50 epochs. The blue line corresponds to the training loss, exhibiting an initial steep decrease, demonstrating rapid learning during the earliest phases of model training. Subsequently, the data stabilizes, indicating that the model is approaching a point of minimal loss.

The orange line, which represents the validation loss, exhibits more volatility. The variability observed may signify that the model is facing challenges in effectively applying its learned knowledge to the validation dataset. This might potentially indicate overfitting if the pattern persists or becomes more pronounced. Nevertheless, given there is no significant rise in validation loss over time, it seems that the model can maintain its capacity to generalize rather well. The objective is to reduce the disparity between the training and validation loss, which signifies a model that exhibits effective learning and generalization capabilities towards novel, unobserved input.

Figure 10 illustrates the progression of a model’s training in categorizing data into three distinct groups. The training loss, shown by the blue line, exhibits a rapid decline followed by a period of stability. This characteristic pattern indicates that the model efficiently acquires knowledge from the training data. The test loss, shown by the orange line, declines in parallel with the training loss but displays intermittent variations, which may be attributed to the model’s performance on the validation dataset. The graph demonstrates a learning process in which the model effectively adapts to the training data and maintains a satisfactory performance on the validation data that has not been previously observed. This pattern is beneficial as it indicates that the model has successfully acquired generalizable patterns from the data without suffering from overfitting.

Figure 11 illustrates the progression of training loss and validation loss during 50 epochs during the training of a machine learning model. The training loss, shown by the blue line, exhibits a sharp decrease, suggesting fast acquisition of knowledge, and then stabilizes, indicating that the model has begun to reach a state of convergence. Nevertheless, the validation loss (shown by the orange line) displays instability and a notable sudden increase, suggesting the presence of an abnormality in the validation data or the possibility of overfitting at that particular time. However, the validation loss consistently decreases, indicating that the model maintains good generalization to new data even after the first spike. Such training aims to ensure that the validation loss drops in parallel with the training loss, reducing the disparity between them. This indicates a model consistently performing well on familiar and unfamiliar data.

Figure 12 illustrates the loss measure of a deep learning model throughout 50 epochs. The blue line shows the training loss offers a substantial decline after the early epochs, indicating the model’s rapid learning progress. Subsequently, it reaches a stable state, suggesting the model is approaching a minimum loss. The test loss, shown in orange, initially shows a similar drop but is distinguished by intermittent spikes that indicate fluctuations in the model’s performance on the validation set. Following spikes, the test loss becomes stable, although it does not reach the same level as the training loss. This implies that the model may not effectively adapt to new data, indicating a potential lack of generalization. This may mean more adjustments to enhance the model’s ability to apply to various situations or examine the data to comprehend the cause of instability.

Furthermore, prec, rec, and f1 score for each class classification are also calculated as follows [58]:

1. *Precision (Prec)*: This is another model performance evaluation metric employed in this study. It can be defined as the quantification of the “True Positive” (TP) results predicted by the model. It is expressed as:

9 $$Precision= \frac{TP}{TP+FP} \times 100\%$$

where TP signifies true positive and FP signifies false positive

2. *Recall (Sensitivity):* Recall is the metric that measures how sensitive a predictive model performs. It measures the proportion of the positive instances that are truly predicted as positive. It is usually expressed as:where TP implies true positive, and FN signifies false negative
  10 $$Recall= \frac{TP}{TP+FN} \times100$$

3. *F1-Score:* F1-Score is a metric that expresses stability between Prec and Rec. It is the harmonic average of both Rec and Prec.
  F1-score is expressed as:

11 $$F1\ score=2 \times \frac{Precision \times Recall}{Precision+Recall}$$

The average prec, rec, and f1 scores for each class classification are obtainable in Table 6. Table 6 presents a concise overview of the performance indicators of the PulmoNet model in several categorization situations. The model demonstrates a notable equilibrium between accuracy (94%) and recall (95.01%) in the 4-class classification, indicating its proficiency in accurately detecting affirmative instances within the four categories. The model has a commendable accuracy (90.52%) and F1 score (88.65%), suggesting its strong performance. However, there is potential for improvement in achieving a better equilibrium between precision and recall.

**Table 6:** Average acc, prec, rec, and f1 scores for each class classification

Model classification	ACC (%)	PREC	REC	F1 Score
4 Class	94	90.52	95.01	88.65
3 Class	95.40	95.15	98.54	97.34
2 Class (Covid_19 and Healthy)	99.4	98.86	99.41	98.46
2 Class (Pneumonia and Healthy)	98.3	99.12	99.01	99.24

In the case of the 3-class scenario, the model demonstrates enhancement in all measures, notably achieving a recall of 98.54% and an F1 score of 97.34%. These results indicate a remarkable level of model sensitivity and a well-balanced precision-recall trade-off.

The model performs exceptionally in binary classifications, with accuracy, precision, recall, and F1 scores over 98%. This implies that when the model is given the duty of differentiating between just two categories, its capacity to recognize and categorize each one accurately is quite dependable.

**Fig. 13:**
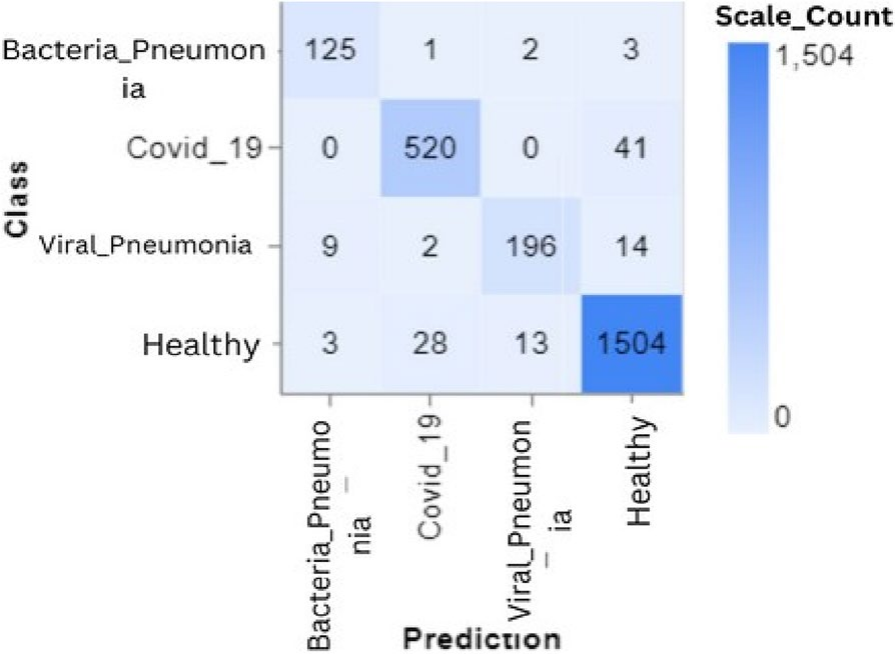
Confusion matrix for 4 class classification

Figure 13 visually illustrates the accuracy of the PulmoNet model in categorizing instances as Bacterial Pneumonia, COVID-19, Viral Pneumonia, or Healthy. The matrix displays the quantity of forecasts generated by the model compared to the actual categories. Specifically, the cases anticipated to be Bacterial Pneumonia 125 were accurately diagnosed, whereas a small number of cases were mistakenly labeled as Covid_19, Viral Pneumonia, or Healthy. Likewise, a significant proportion of Covid_19 cases were correctly categorized, although there was some ambiguity in distinguishing them from healthy instances. Some misdiagnosis is seen in cases of Viral Pneumonia, especially regarding Healthy individuals. The Healthy class has the most significant number of accurate classifications, showing a robust capability of the model to identify persons in good health, but with some occasional misunderstanding with other illness states. The matrix is essential for comprehending the model’s diagnostic capabilities and identifying areas needing refinement.

**Fig. 14:**
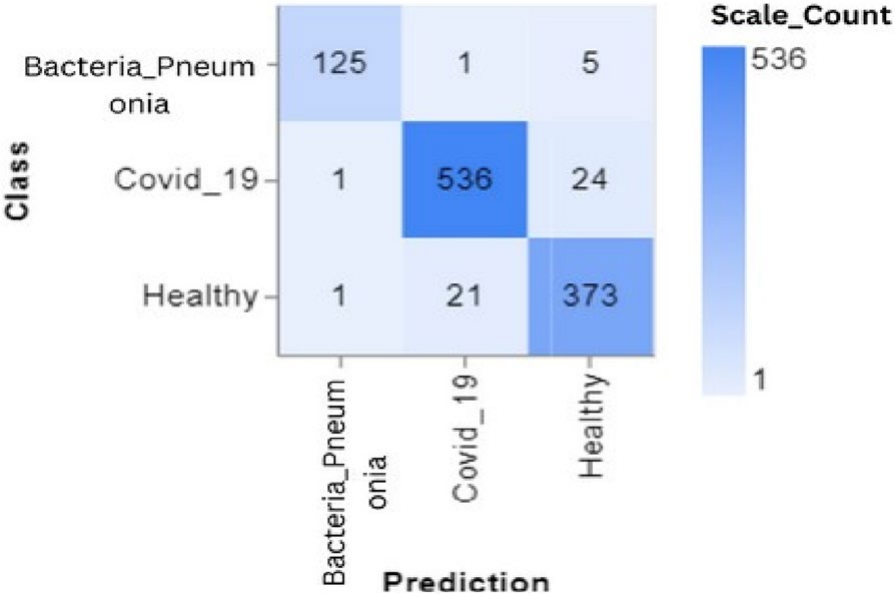
Confusion matrix for 3 class classification

The confusion matrix shown in Fig. 14 illustrates the model’s efficacy in differentiating between the Bacterial Pneumonia, Covid-19, and Healthy classes in a 3-class classification scenario. The matrix demonstrates high precision in categorizing Covid-19, with 536 accurate forecasts and only a minimal number of instances misclassified as Bacterial Pneumonia or Healthy. Bacterial Pneumonia has a high accuracy rate of categorization (125 out of 131) but with significant misclassification in the Healthy group. The Healthy class has a high level of accuracy, with 373 correct predictions. However, there are a few situations when Healthy occurrences are incorrectly labelled as Bacterial Pneumonia or Covid-19. This graphic facilitates comprehension of the model’s diagnostic precision and may inform enhancements in the accuracy of predictions for certain classes.

**Fig. 15:**
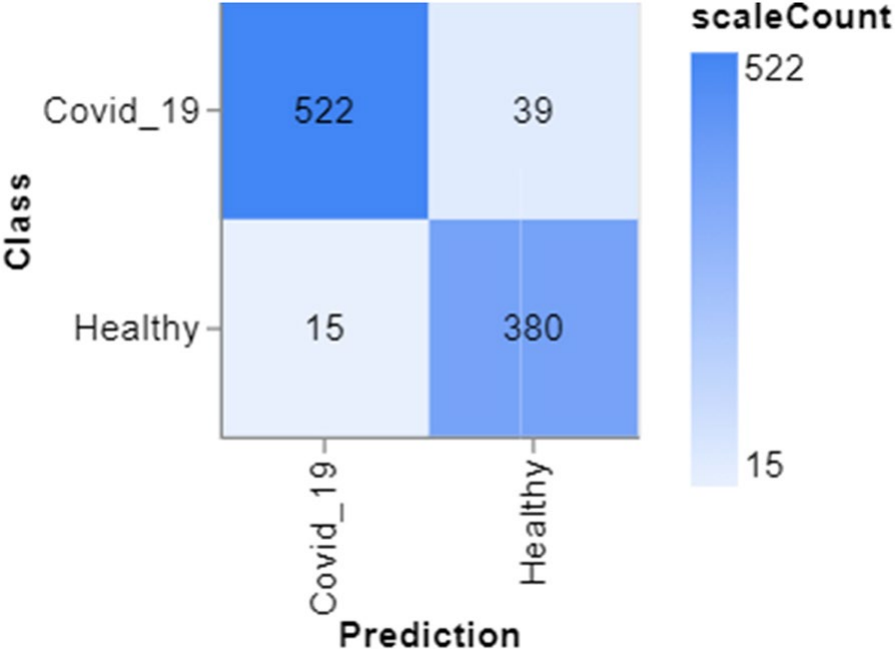
Confusion matrix for 2 class classifications (Covid_19 and Healthy)

Figure 15 displays the results of categorizing samples into either the COVID-19 or Healthy classes. The dark blue squares on the diagonal indicate accurate categorizations, consisting of 522 instances correctly identified as COVID-19 positive and 380 samples correctly identified as Healthy negative. The lighter blue squares indicate misclassifications, composed of 39 false negatives, where COVID-19 patients were erroneously categorized as Healthy, and 15 false positives, where Healthy cases were wrongly recognized as COVID-19. The elevated values along the diagonal indicate a robust model efficacy in differentiating COVID-19 patients from healthy persons.

**Fig. 16:**
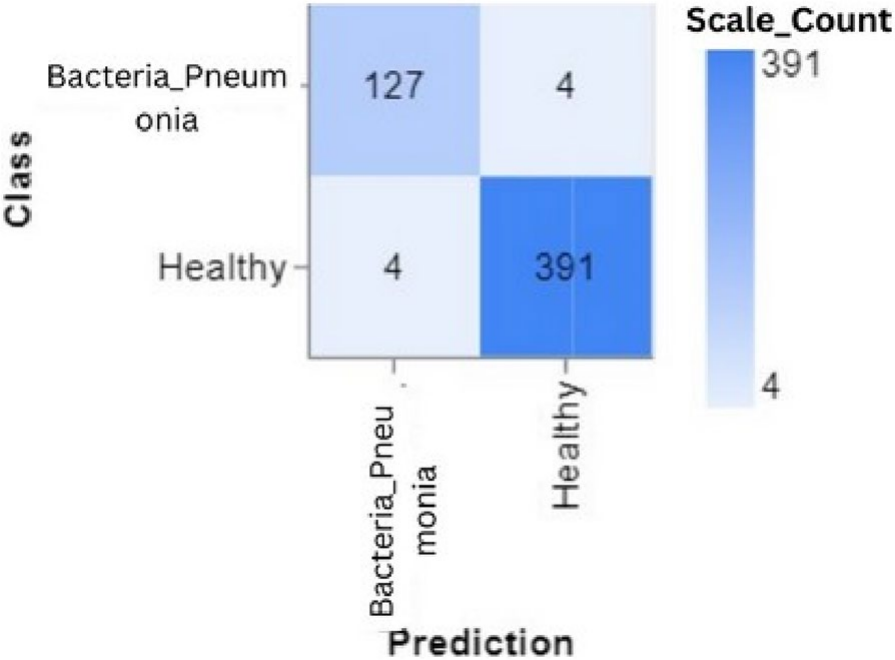
Confusion matrix for 2 class classifications (Pneumonia and Healthy)

Figure 16 illustrates a confusion matrix for a binary classification issue that distinguishes between instances of pneumonia and healthy individuals. The matrix accurately identifies 127 pneumonia and 391 cases of healthy controls by the model, as seen by the substantial numbers along the diagonal from the top left to the bottom right. The occurrence of misclassification is minimal, with a mere four instances of pneumonia being erroneously labelled as healthy and four healthy cases misclassified as pneumonia. The matrix exhibits notable accuracy and precision in the model’s predictions, demonstrating a well-balanced performance across both classes.

Figures 13, 14, 15 and 16 shows the confusion matrix of the classifications such as 4 class, 3 class, and 2 classes. In summary, the suggested technique realized an average accuracy of 94%, 95%, 95%, and 98% for 4 classes, 3 classes, 2 classes (Covid_19 and Healthy), and 2 classes (Pneumonia and Healthy) classifications. Prec, rec, and f1 score values recorded for each class show good performance of the model as a higher recall value indicates an encouraging low false negative case.

Experimental outcomes attained by the suggested technique are further likened to some selected related research conducted in the past. This is done to validate further the experimental outcomes realized by our model. A summary of the comparative investigation is obtainable in Table 7.

**Table 7:** Comparative examination of the performance of PulmoNet with other selected models

Models	4 class				3 class				2 class (Covid_19 and Healthy)				2 classes (Pneumonia and Healthy)				No of samples			
**Metrics**	**ACC**	**PREC**	**REC**	**F1 SCORE**	**ACC**	**PREC**	**REC**	**F1 SCORE**	**ACC**	**PREC**	**REC**	**F1 SCORE**	**ACC**	**PREC**	**REC**	**F1 SCORE**	**BACT_PNEUMONIA**	**COVID_19**	**HEALTHY**	**VIRAL PNEUMONIA**
(Khan et al. [53]	89.6	90	89.92	89.8	95	95	96.9	95.6	99	98.3	99.3	98.5	NA	NA	NA	NA	330	284	310	327
(Hussain et al. [59]	91.2	91.13	90.61	92.17	94.2	93.82	93.82	94.67	99.1	98.76	97.66	97.93	**NA**	**NA**	**NA**	**NA**	400	500	800	400
PulmoNet	94	90.52	95.01	88.65	95.4	95.15	98.54	97.34	99.4	98.86	99.41	98.46	98.3	99.12	99.01	99.24	880	3,749	10,320	1,478

Table 7 displays a comparative examination of performance measures for different models in various class classification tasks. PulmoNet demonstrates exceptional performance, especially in the 2-class classifications for COVID-19/Healthy and Pneumonia/Healthy, achieving accuracies of 98%. This demonstrates PulmoNet’’s particular aptitude in binary categorizations. Furthermore, it shows strong competitiveness in multi-class settings, exhibiting performance measures equivalent or superior to those of other models. This suggests its resilience and ability to generalize. This comparison highlights the potential of PulmoNet as a dependable tool in clinical settings for diagnosing lung illnesses.

As presented in the table, the performance accuracy of Khan et al., [53] is 89.6% for 4 classes, 95% for 3 classes, and 99% for two classes (covid_19 and healthy) classifications and (Hussain et al. [59] recorded 91.2%, 94.2%, and 99.1% respectively for 4 class, 3 class, and 2 class (covid_19 and healthy) classifications compared to 94%, 95.4%, 99.4% obtained by PulmoNet for 4 class, 3 class, and 2 class (covid_19 and healthy) classifications. Another point worthy of mention here is the second 2 class (pneumonia and healthy) classification which is not reported by both Khan et al., [53] or Hussain et al., [59] in their works. This class classification recorded an accuracy of 98.3% in this work. Finally, higher precision, recall, and f1 score recorded by our work in most class classifications indicate better performance by our model.

### The study key findings

The study on the PulmoNet model represents a significant advancement in the application of artificial intelligence for the diagnosis of pulmonary diseases. The model’s performance was rigorously evaluated across various classification scenarios, yielding insightful results that demonstrate its efficacy and potential clinical applicability.

Performance in multi-class and binary classifications:

- *4-Class Configuration *(Table 2): PulmoNet exhibited high diagnostic accuracy across different pulmonary diseases and healthy states. Specifically, it achieved 95% accuracy for bacterial pneumonia, 93% for COVID-19, 91% for viral pneumonia, and an impressive 97% for healthy cases. These results underscore the model’s capability to accurately distinguish between complex disease categories, a crucial requirement in clinical diagnostics.
- *3-Class Configuration *(Table 3): The model maintained consistent performance, particularly showing improvement in COVID-19 detection (96% accuracy). This indicates the model’s adaptability and precision in scenarios with fewer classification categories.
- *Binary Classifications *(Tables 4 and 5): In binary classifications, PulmoNet demonstrated exceptional accuracy, especially notable in distinguishing COVID-19 (99% accuracy) and healthy cases (99.8% accuracy). Such high performance in binary classifications suggests the model’s potential utility in preliminary screening and rapid diagnostics.

Comparative Analysis with Existing Models:

- *Comparative Evaluation (*Table 7*):* When compared with other studies (Khan et al., Hussain et al.), PulmoNet showed competitive or superior performance in several aspects. This comparison not only validates the effectiveness of PulmoNet but also positions it as a potential benchmark in the field of AI-driven pulmonary disease detection.

Training and Validation Insights:

- *Model Training Dynamics *(Figs. 5, 6, 7 and 8): The accuracy and loss graphs for each class configuration provided insights into the model’s training dynamics. The close alignment of training and validation accuracy indicates that PulmoNet is well-trained, effectively learning from the training data without overfitting.
- *Confusion Matrix Analysis *(Figs. 13, 14, 15 and 16): The confusion matrices for each classification task offer a detailed view of the model’s diagnostic accuracy, illustrating its strengths in correctly identifying cases and areas where improvements could be made.

Clinical Implications and Future Directions:

- *Clinical Relevance:* The high accuracy and reliability of PulmoNet suggest its significant potential in clinical environments for enhancing diagnostic procedures for pulmonary diseases.
- *Future Enhancements:* Acknowledging the limitations regarding dataset diversity and size, future studies are planned to test the model on a larger and more varied dataset. This will help in further validating the model’s robustness and generalization capabilities.

In summary, the PulmoNet model, through this study, demonstrates promising capabilities in revolutionizing AI-driven illness diagnosis, particularly in the realm of pulmonary diseases. Its high accuracy across various class configurations and strong performance compared to existing models highlight its potential as a valuable tool in medical diagnostics. Future enhancements and broader clinical testing are anticipated to further solidify its applicability and effectiveness in healthcare settings.

## Discussion

The PulmoNet model demonstrated exceptional performance in a 4-class classification task, with accuracies of 97% for healthy cases, 95% for bacterial pneumonia cases, 93% for COVID-19 cases, and 91% for viral pneumonia cases. The accuracies for the three classes in a 3-class configuration were as follows: 95.3% for healthy, 96% for COVID-19, and 95% for bacterial pneumonia. The model attained a 99% accuracy for classifying COVID-19 cases and a 99.8% accuracy for classifying healthy cases in a 2-class classification task, demonstrating its proficiency in binary classifications. The measurements, including accuracy, recall, and f1-scores, highlight the promise of PulmoNet in revolutionizing AI-driven illness diagnosis and its suitability in clinical environments.

The PulmoNet model demonstrated remarkable precision in identifying lung illnesses in different categorization circumstances. The system’s performance in a 4-class configuration was outstanding, with excellent accuracy rates for each illness category, notably excelling in binary classifications. The model’s resilience was further validated by crucial performance indicators such as accuracy, recall, and f1-score, vital for minimizing incorrect diagnoses in medical environments.

PulmoNet exhibited more incredible skills than current models, indicating the potential to redefine AI-driven pulmonary illness identification benchmarks. The training and validation procedure, as shown by the balanced accuracy and loss graphs and confusion matrices, suggested a proficiently trained model without any problems of overfitting or underfitting.

These findings emphasize the significant influence of artificial intelligence (AI) and deep learning in medical diagnosis, especially for pulmonary illnesses. The high accuracy and dependability of PulmoNet make it very applicable in clinical settings, where it can potentially improve diagnostic processes and patient outcomes. Potential advancements may enhance the model, expand its clinical use, and enhance healthcare diagnostics.

## Limitations of the study

The study’s limitation mostly pertain to the dataset and methodology. The dataset, sourced from many public sources, may lack the necessary diversity and size to guarantee the model’s generalisation capacity across different populations. Furthermore, the model explicitly targets three distinct lung disorders, potentially restricting its relevance to other respiratory ailments. The exclusive dependence on X-ray and CT scan pictures may also provide problems since these imaging modalities include inherent limits. Additionally, the research necessitates clinical validation to evaluate the real-time applicability and efficacy in realistic healthcare environments. Prospects include investigating picture segmentation and hyperparameter adjustment to improve accuracy.

## Conclusions

Ever since the COVID-19 pandemic broke out in late 2019, pulmonary diseases—which are already the third biggest killer on Earth—have been a major problem for public health throughout the planet. Because of this, finding reliable diagnostic tools quickly is more important than ever. This paper presents PulmoNet, a state-of-the-art deep learning (DL) model that uses radiographic images like X-rays and CT scans to improve the diagnosis of a range of pulmonary disorders, including bacterial pneumonia, COVID-19, and viral pneumonia.

A dataset consisting of thousands of photos taken from freely accessible databases was heavily used to build PulmoNet—the extensive dataset allowed for thorough model training, guaranteeing its efficacy in many diagnostic situations. Compared with two other popular methods, PulmoNet performed far better in accuracy, precision, recall, and f1-score. A promising diagnostic tool, PulmoNet showed remarkable competence in both multi-class and binary classifications.

The relevance of PulmoNet is that it can provide healthcare providers with a significant tool for understanding lung disorders, including COVID-19, and making correct diagnoses promptly. Offering a solution to the urgent demand for effective illness detection techniques in the face of recurring health crises, its excellent accuracy in diagnosing numerous lung disorders represents a considerable contribution to medical diagnostics.

The research finds several ways to improve PulmoNet’s effectiveness in the future. To further enhance accuracy in 4 and 3 class setups, future research will concentrate on investigating supplementary approaches like as picture segmentation and hyperparameter tuning. Plans are also in the works for the model’s specialist clinical testing and validation, which will guarantee its dependability and practicality. These procedures are essential for bringing the model into practical use, where it may greatly assist in dealing with the difficulties caused by lung disorders. As a result of these planned developments, PulmoNet should become an even more effective weapon in the fight against pulmonary disorders, which should lead to improved healthcare results on a worldwide scale.

In the future we have proposed to test our model on a more extensive dataset. This will help to further evaluate the model’s generalization capabilities and ensure that it maintains high accuracy and reliability when exposed to a broader range of data. We have also propose re-evaluating the models using a standard dataset or delineating the differences in test sets and settings when discussing the results.

## Data Availability

The data that support the findings of this study are available on Kaggle. The data can also be accessed at https://www.kaggle.com/datasets/abdulahiabdulrahman/pulmonet-dataset.
